# Design of High‐Performance Organic Nonlinear Optical and Terahertz Crystals by Controlling the van der Waals Volume

**DOI:** 10.1002/advs.202304767

**Published:** 2023-10-22

**Authors:** Bong‐Rim Shin, Uros Puc, Yu‐Jin Park, Dong‐Joo Kim, Chae‐Won Lee, Woojin Yoon, Hoseop Yun, Chaeyoon Kim, Fabian Rotermund, Mojca Jazbinsek, O‐Pil Kwon

**Affiliations:** ^1^ Department of Molecular Science and Technology Ajou University Suwon 16499 South Korea; ^2^ Institute of Computational Physics Zurich University of Applied Sciences (ZHAW) Winterthur 8401 Switzerland; ^3^ Research Institute of Basic Sciences Department of Chemistry Department of Energy Systems Research Ajou University Suwon 16499 South Korea; ^4^ Department of Physics Korea Advanced Institute of Science and Technology (KAIST) Daejeon 34141 South Korea

**Keywords:** crystal structure control, nonlinear optics, organic crystals, terahertz photonics, van der Waals volume

## Abstract

In the development of new organic crystals for nonlinear optical and terahertz (THz) applications, it is very challenging to achieve the essentially required non‐centrosymmetric molecular arrangement. Moreover, the resulting crystal structure is mostly unpredictable due to highly dipolar molecular components with complex functional substituents. In this work, new organic salt crystals with top‐level macroscopic optical nonlinearity by controlling the van der Waals volume (*V*_vdW_), rather than by trial and error, are logically designed. When the *V*_vdW_ of molecular ionic components varies, the corresponding crystal symmetry shows an observable trend: change from centrosymmetric to non‐centrosymmetric and back to centrosymmetric. All non‐centrosymmetric crystals exhibit an isomorphic *P*1 crystal structure with an excellent macroscopic second‐order nonlinear optical response. Apart from the top‐level macroscopic optical nonlinearity, new organic crystals introducing highly electronegative fluorinated substituents with strong secondary bonding ability show excellent performance in efficient and broadband THz wave generation, high crystal density, high thermal stability, and good bulk crystal growth ability.

## Graphical abstract

A rational design of organic salt crystals for high‐performance nonlinear optical and THz applications is successfully demonstrated. The associated crystal structure reliably varies from centrosymmetric to non‐centrosymmetric and then again to centrosymmetric by controlling the van der Waals volume of the molecular ionic components.

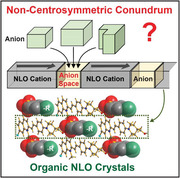

## 1 Introduction

Organic nonlinear optical crystals are highly attractive for a variety of nonlinear optical and terahertz (THz) photonic applications.^[^ ^1^, ^2^, ^3^, ^4^, ^5^, ^6^, ^7^, ^8^, ^9^, ^10^ ^]^ Compared to inorganic crystals, many organic crystals exhibit a superior second‐order nonlinear optical coefficient when highly nonlinear optical chromophores assemble in a non‐centrosymmetric structure with a high‐order parameter (i.e., close to parallel alignment of chromophores in the crystalline state).^[^ ^11^, ^12^, ^13^ ^]^ This superior macroscopic optical nonlinearity of organic crystals generally leads to a significantly higher optical‐to‐THz conversion efficiency, which is often accompanied by a broader THz spectral bandwidth,^[^ ^6^, ^13^, ^14^, ^15^, ^16^, ^17^, ^18^, ^19^ ^]^ supported by better phase matching conditions and weaker absorption resonances in the THz frequency range.^[^ ^7^, ^20^ ^]^

Organic nonlinear optical crystals consist of molecular components including highly polar nonlinear optical chromophores. The ability to chemically modify organic crystals is usually regarded as an advantage to achieve the desired physical as well as diagonal and off‐diagonal nonlinear optical characteristics.^[^ ^20^ ^]^ Unfortunately, however, in organic crystals based on highly efficient nonlinear optical chromophores, the crystal structure is usually unpredictable and mostly exhibits a centrosymmetric antiparallel dipole‐dipole aggregation of highly polar chromophores with a high dipole moment, i.e., loss of the macroscopic second‐order optical nonlinearity. Consequently, achieving non‐centrosymmetric molecular order, especially with the desired high‐order parameter in the crystalline state, remains an enigmatic problem. This difficulty makes the design additionally challenging for target applications, such as THz generation in this work. The conundrum of whether a non‐centrosymmetric molecular ordering can be achieved therefore unfailingly raises the question of the possibility of rational design for high‐performance organic nonlinear optical and THz crystals.

**Figure 1:**
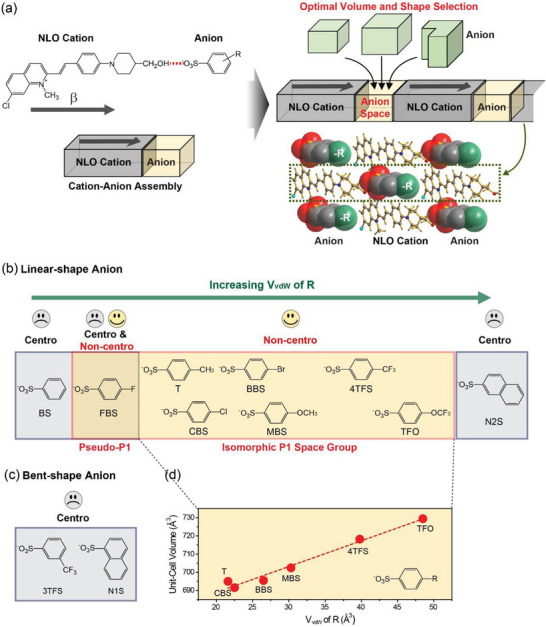
Schematics of a) rational design of non‐centrosymmetric molecular ordering in the crystalline state, based on the head‐to‐tail, series‐type cation‐anion assembly between the highly nonlinear optical PM7ClQ cationic chromophore and various counter anions having different volume and shape; b) linear‐ and c) bent‐shape anions with the resulting crystal structure (Non‐Centro: non‐centrosymmetric and Centro: centrosymmetric crystal structure). d) Unit‐cell volume of isomorphic crystals with *P*1 crystal symmetry (*Z* = 1) as a function of van der Waals volume, *V*_vdW_ of R substituents^[^ ^29^, ^30^ ^]^ on linear‐shape anions; the dotted line has a slope of 1.41.

In this work, we successfully demonstrate a rational design of new organic salt crystals possessing top‐level macroscopic optical nonlinearity by controlling the van der Waals volume and shape of molecular components. In addition, highly electronegative fluorinated substituents with strong secondary‐bond capability are introduced for targeted THz generation applications. In organic salt crystals, the molecular components (cations and anions) mostly favor a tight packing without void (or smallest void). Consequently, the molecular packing of cations and anions satisfying charge balance might have a certain rule for their volume and shape; like a puzzle that requires the correct selection of its shape and size (**Figure** **1**a). In this work, controlling the van der Waals volume and shape of molecular ionic components is used as a design strategy for new organic nonlinear optical salt crystals. Interestingly, the variation in van der Waals volume and shape of molecular ionic components shows an observable trend in crystal symmetry, which changes from centrosymmetric to non‐centrosymmetric and then back to centrosymmetric. All newly designed non‐centrosymmetric crystals with fluorinated substituents exhibit the *P*1 space group with the ideal order parameter and top‐level second‐order optical nonlinearity and exhibit excellent THz generation performance with distinguishable THz characteristics. Therefore, controlling the van der Waals volume and shape of molecular components is a potential strategy for developing high‐performance organic nonlinear optical and THz crystals.

## 2 Results and Discussion

### 2.1 Design with Control of van der Waals Volume and Shape

For designing new organic salt crystals possessing top‐level macroscopic second‐order optical nonlinearity, molecular components, cations, and anions are introduced for different functions in the crystalline state, molecular optical nonlinearity, and charge balance with non‐centrosymmetric and tight‐packing alignment, respectively. The cation, 7‐chloro‐2‐(4‐(4‐(hydroxymethyl)piperidin‐1‐yl)styryl)−1‐methyl‐quinolinium (PM7ClQ, Figure 1a) was chosen due to its large molecular optical nonlinearity (large first hyperpolarizability *β*) and large isomorphic tolerance space in the crystalline state.^[^ ^21^, ^22^ ^]^

For anions various aromatic sulfonates, benzenesulfonates, and naphthalene sulfonates (Figure 1b,c) are introduced. The sulfonate group on anions can form head‐to‐tail cation‐anion assembly by a strong hydrogen bond (─OH···^−^O_3_S─) presented with the red dotted line in Figure 1a. In addition, the introduced anions can act as a matchmaker to achieve a non‐centrosymmetric and tight packing alignment. In many nonlinear optical organic salt crystals with top‐level macroscopic optical nonlinearity, the volume ratio between cations and anions is very narrow.^[^ ^13^ ^]^ For example, stilbene‐type chromophores, 4‐(4‐(dimethylamino)styryl)−1‐methylpyridinium (DAS), 2‐(4‐hydroxy‐3‐methoxystyryl)−1‐methylquinolinium (HMQ), 2‐(4‐hydroxy‐3‐methoxystyryl)−3‐methylbenzothiazol‐3‐ium (HMB), 2‐(4‐(4‐(hydroxymethyl)piperidin‐1‐yl)styryl)−3‐methylbenzothiazol‐3‐ium (PMB) and 1‐ethyl‐2‐(4‐(4‐(hydroxymethyl)piperidin‐1‐yl)styryl)−3,3‐dimethyl‐3H‐indolium (EHPSI) cations and their analogous cations exhibit a high order parameter when crystallized with benzenesulfonate (and naphthalene sulfonate) anions having a single phenyl (and naphthalene) ring.^[^ ^14^, ^23^, ^24^, ^25^, ^26^, ^27^, ^28^ ^]^ Using relatively bigger anions such as stilbene‐type 4‐((4‐(dimethylamino)phenyl)diazenyl)benzenesulfonate) (MO), these cations are centrosymmetrically aligned in crystals.^[^ ^23^ ^]^ In contrast, the 4‐(4‐(4‐(hydroxymethyl)piperidin‐1‐yl)styryl)−1‐(pyrimidin‐2‐yl)pyridin‐1‐ium (PMPR) cation, which is larger than the above‐mentioned cations, exhibits a high order parameter when crystallized with bigger stilbene‐type MO anions.^[^ ^13^ ^]^ These results suggest that optimal selection of the size (and volume) of the anions for a given cation is required for non‐centrosymmetric and tight packing alignment in the crystalline state.

In this work, we examined two types of aromatic sulfonate anions; linear‐shape anions (Figure 1b) and bent‐shape anions (Figure 1c). In addition, we varied the van der Waals volume, *V*_vdW_ of anions. In Figure 1b,c, the van der Waals volume, *V*_vdW_ of R substituents increases from left to right.^[^ ^29^, ^30^, ^31^ ^]^ The PM7ClQ‐based crystals with linear‐shape benzenesulfonate (BS), 4‐fluorobenzenesulfonate (FBS), 4‐(trifluoromethyl)benzenesulfonate (4TFS), 4‐(trifluoromethoxy)benzenesulfonate (TFO), and naphthalene‐2‐sulfonate (N2S) anions, and bent‐shape 3‐(trifluoromethyl)benzenesulfonate (3TFS), and naphthalene‐1‐sulfonate (N1S) anions were newly designed and synthesized. The linear‐shape 4‐methylbenzenesulfonate (T), 4‐chlorobenzenesulfonate (CBS), 4‐bromobenzenesulfonate (BBS), and 4‐methoxybenzenesulfonate (MBS) anions were previously studied in combination with the PM7ClQ cation.^[^ ^21^, ^22^ ^]^

By controlling their van der Waals volume, *V*_vdW_ of anions with different shapes, highly electronegative fluorinated substituents that can form strong secondary bonds^[^ ^32^, ^33^ ^]^ are incorporated into anions in new PM7ClQ‐based crystals; PM7ClQ‐FBS, PM7ClQ‐4TFS, PM7ClQ‐3TFS and PM7ClQ‐TFO. The fluoro (─F), trifluoromethyl (─CF_3_), and trifluoromethoxy (─OCF_3_) groups for FBS, TFS, and TFO anions, respectively, having different conformational shapes^[^ ^34^ ^]^ and different numbers of F atoms may form hydrogen bonds (e.g., F⋅⋅⋅H) with different strength and direction to PM7ClQ cations (and anions), which may affect physical and THz characteristics.

### 2.2 Crystal Symmetry with Top‐Level Optical Nonlinearity

To screen whether the newly designed PM7ClQ‐based crystals possess non‐centrosymmetric or centrosymmetric molecular order, qualitative powder second harmonic generation (SHG) measurements^[^ ^35^ ^]^ were performed using excitation pulses at 1300 nm. As summarized in Figure 1b, in a certain range of van der Waals volumes of linear‐shape anions (FBS, 4TFS, and TFO, yellow highlighted area), the corresponding crystals exhibited a strong SHG signal at 650 nm. In contrast, crystals with smaller or bigger anions (BS and N2S, gray highlighted area) did not generate a measurable SHG signal. With both investigated bent‐shape anions (3TFS and N1S, Figure 1c), SHG signals were not observed. It is noticeable that linear‐shape anions within a relatively narrow range of van der Waals volumes (*V*_vdW_ of R substituents of ≈13–49 Å) result in a non‐centrosymmetric molecular arrangement of PM7ClQ cations in the crystalline state.

To investigate the crystal structure of the newly developed non‐centrosymmetric PM7ClQ‐FBS, PM7ClQ‐4TFS, and PM7ClQ‐TFO, single crystals were grown by solution‐cooling methods. The details are described in Sections B and C (SupportingInformation). The obtained PM7ClQ‐4TFS and PM7ClQ‐TFO single crystals exhibit a non‐centrosymmetric *P*1 crystal space group. PM7ClQ‐FBS exhibit two polymorphs; non‐centrosymmetric PM7ClQ‐FBS (Phase‐I) and centrosymmetric PM7ClQ‐FBS (Phase‐II). Interestingly, both PM7ClQ‐4TFS and PM7ClQ‐TFO crystals exhibit an isomorphic crystal structure with benchmark PM7ClQ‐T crystals (**Table** **1**). The summary of crystallographic parameters and physical properties of these crystals is listed in Table 1. Also, PM7ClQ‐FBS (Phase‐I) crystals may exhibit an isomorphic or at least a pseudo‐isomorphic crystal structure to PM7ClQ‐T crystals (see Section C, Supporting Information).

**Table 1:** Summary of physical properties and crystal characteristics of, PM7ClQ‐4TFS, PM7ClQ‐TFO, and PM7ClQ‐T crystals.

	PM7ClQ‐TFO	PM7ClQ‐4TFS	PM7ClQ‐T^[^ ^21^ ^]^
*T*_m_ (°C)	255	258	256
solubility at 40 °C (g per 100 g MeOH, MeOH:AcCN)	2.31, 3.82	1.23, 2.11	0.95, 1.59
space group	triclinic *P*1	triclinic *P*1	triclinic *P*1
molecular weight	635.08	619.08	565.1
Z	1	1	1
*V* (Å^3^)	729.41	718.20	694.99
cell parameters	*a* = 7.0759 (4) Å *b* = 10.3428 (9) Å *c* = 10.8490 (7) Å *α* = 71.427 (3)° *β* = 76.025 (1)° *γ* = 82.894 (3)°	*a* = 7.0905(5) Å *b* = 10.3016(7) Å *c* = 11.0434(9) Å *α* = 66.469(2)° *β* = 76.744(1)° *γ* = 81.248(2)°,	*a* = 7.0061(3) Å *b* = 10.0242(5) Å *c* = 11.0610(5) Å *α* = 67.484(1)° *β* = 75.747(1)° *γ* = 82.584(1)°
density(g cm^−3^)	1.446	1.431	1.350
void volume (%)	29.4	29.2	29.4
order parameter cos^3^ *θ*_p_	1.0	1.0	1.0
square root of SHG intensity relative to PM7ClQ‐T^a)^	0.95 ± 0.05	0.96 ± 0.04	1.0
effective first hyperpolarizability $\beta_{iii}^{\mathrm{eff}}$ (10^−30^ esu)	248	251	261

^a)^ average at 1800 nm using pump power of 10 and 20 mW (Figure 2e).

Consequently, as summarized in Figure 1b, when increasing the van der Waals volume of linear‐shape anions with the identical PM7ClQ cation, the crystal symmetry varies from centrosymmetric with the smallest anion (BS) to non‐centrosymmetric (pseudo‐)isomorphic *P*1 crystal structure with intermediate size (FBS) that also exhibits a centrosymmetric polymorph. With further increase of the anion volume (T, CBS, BBS, MBS, 4TFS, and TFO), but within the optimal range of van der Waals volume (*V*_vdW_ of R substituents of ≈13–49 Å), all 6 crystals exhibit an isomorphic non‐centrosymmetric *P*1 crystal structure. Using the anion N2S which is larger than the optimal volume, the corresponding crystal again shows centrosymmetric symmetry.

The 6 isomorphic crystals (PM7ClQ‐T, PM7ClQ‐CBS, PM7ClQ‐BBS, PM7ClQ‐MBS, PM7ClQ‐4TFS, and PM7ClQ‐TFO) exhibit a series‐type cation‐anion assembly^[^ ^20^ ^]^ and the volume of the unit‐cell (*Z* = 1) relates to van der Waals volume, *V*_vdW_ of R substituents on anions. As shown in Figure 1d, the unit‐cell volume of isomorphic crystal structure is approximately linearly proportional to van der Waals volume, *V*_vdW_ of R substituents.

**Figure 2:**
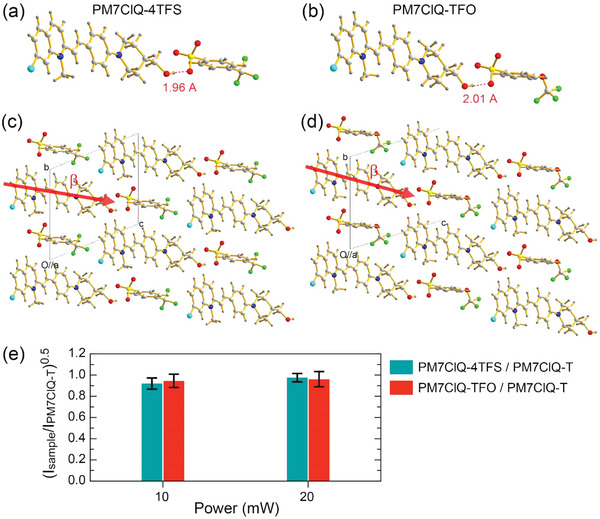
Head‐to‐tail cation‐anion assembly based on the strongest hydrogen bond (dotted red line) in a) PM7ClQ‐4TFS and b) PM7ClQ‐TFO crystals and c and d) their molecular ordering with non‐centrosymmetric *P*1 space group symmetry (*Z* = 1). e) Square root of the SHG intensity of PM7ClQ‐4TFS (PM7ClQ‐TFO) relative to PM7ClQ‐T in powder SHG measurements at non‐resonant fundamental wavelength of 1800 nm (pulse duration of 130 fs and repetition rate of 1 kHz) and average pump power of 10 and 20 mW (average value and the error range for three measured samples).

The non‐centrosymmetric PM7ClQ‐4TFS and PM7ClQ‐TFO crystals, that are newly designed in this work, are suitable for second‐order nonlinear optical and THz applications. This is because of the optimal molecular ordering for a very large macroscopic optical nonlinearity, strong interionic interactions, and excellent bulk crystal growth ability as discussed below. **Figure** **2**a,b shows the series‐type head‐to‐tail cation‐anion assembly in PM7ClQ‐4TFS and PM7ClQ‐TFO crystals, respectively, which is as predicted in Figure 1a. The head‐to‐tail cation‐anion assembly between the PM7ClQ cation and the corresponding anion is based on a very strong hydrogen bond of the ─OH⋅⋅⋅^−^O_3_S─ groups; 1.96 and 2.01 Å for 4TFS and TFO anions, respectively.

Figure 2c,d shows the molecular ordering in PM7ClQ‐4TFS and PM7ClQ‐TFO crystals. As discussed above, PM7ClQ‐4TFS and PM7ClQ‐TFO crystals exhibit an isomorphic *P*1 crystal structure with PM7ClQ‐T crystals possessing very large diagonal effective hyperpolarizability tensor element $\beta_{\mathrm{iii}}^{\mathrm{eff}}$ of 261 × 10^−30^ esu.^[^ ^21^ ^]^ The order parameter cos^3^ *θ*_p_ for both PM7ClQ‐4TFS and PM7ClQ‐TFO crystals is the maximum possible (cos^3^ *θ*_p_ = 1.0) because *Z* = 1, i.e., all cationic chromophores are aligned perfectly parallel in the crystal lattice (Table 1). Consequently, both PM7ClQ‐4TFS and PM7ClQ‐TFO crystals may also possess large macroscopic second‐order optical nonlinearity.

Using excitation pulses at a non‐resonant wavelength of 1800 nm, qualitative powder SHG measurements were carried out because the difference in intermolecular interactions often leads to a significant difference in macroscopic second‐order optical nonlinearity.^[^ ^25^, ^36^ ^]^ As shown in Figure 2e and listed in Table 1, the square root of the SHG intensity of both PM7ClQ‐4TFS and PM7ClQ‐TFO relative to benchmark PM7ClQ‐T is close to the value of 1.0. The square root of the powder SHG intensity is proportional to the effective value of the nonlinear optical susceptibility.^[^ ^12^ ^]^ Although there may be different additional contributions to the powder SHG efficiency,^[^ ^35^ ^]^ the factors such as phase matching, particle size effect, and microcrystal morphology are likely to be very similar for these crystals with isomorphic structure and similarly prepared powder samples. Consequently, the diagonal effective hyperpolarizability tensor element $\beta_{iii}^{\mathrm{eff}}$ for PM7ClQ‐4TFS and PM7ClQ‐TFO is very large, ≈ ≥245 × 10^−30^ esu. Note that this value is considerably higher than that of the widely used conventional DAST (4‐(4‐(dimethylamino)styryl)−1‐methylpyridinium 4‐methylbenzenesulfonate) crystals (≈161 × 10^−30^ esu).^[^ ^37^ ^]^ Therefore, the newly developed PM7ClQ‐4TFS and PM7ClQ‐TFO crystals exhibit top‐level macroscopic optical nonlinearity.

### 2.3 Strong Interactions and High Crystal‐Density by Fluorine Substituents

Although PM7ClQ‐4TFS and PM7ClQ‐TFO (and PM7ClQ‐T) crystals possess an isomorphic crystal structure and similar nonlinear optical properties, it does not imply that all other physical and THz characteristics are also very similar. The newly designed PM7ClQ‐4TFS and PM7ClQ‐TFO crystals consist of fluorinated anions with trifluoromethyl (─CF_3_) and trifluoromethoxy (─OCF_3_) groups, whereas benchmark PM7ClQ‐T crystals consist of non‐fluorinated anions. The fluorinated ─CF_3_ group on 4TFS and TFO anions can create many secondary bonds with a relatively short distance (i.e., strong hydrogen bonds with δ^−^ charge).^[^ ^32^, ^33^ ^]^ The fluorine‐induced interactions in PM7ClQ‐4TFS and PM7ClQ‐TFO crystals can result in different physical properties compared to non‐fluorinated PM7ClQ‐T crystals. In addition, the conformation of anions in fluorinated PM7ClQ‐4TFS and PM7ClQ‐TFO crystals is different, as shown in Figure 2c,d, i.e., the direction of the ─CF_3_ group with respect to the direction of the cation‐anion assembly is different in these crystals. As a consequence, the direction of the fluorine‐induced interactions, their number, and their strength may be different in PM7ClQ‐4TFS and PM7ClQ‐TFO crystals.

**Figure 3:**
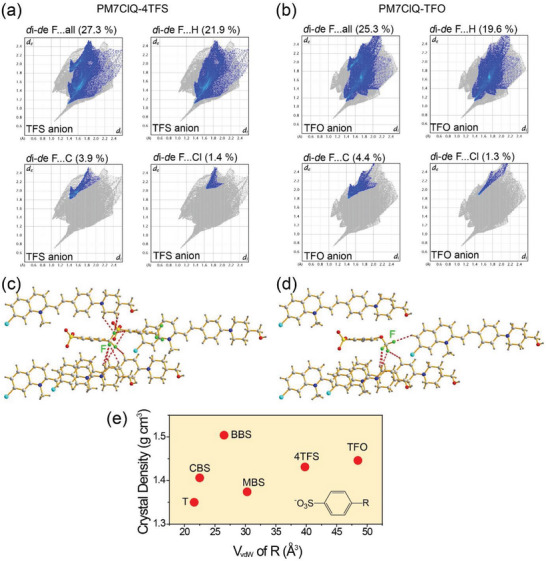
a–d) Fluorine‐induced interionic interactions of (a,c) 4TFS and (b,d) TFO anions on fluorinated PM7ClQ‐4TFS and PM7ClQ‐TFO crystals, respectively: a,b) Hirshfeld surface fingerprints of anions. c,d) Strongest hydrogen bonds with one anion: the red dotted lines present representative F⋅⋅⋅H interactions (distance <3.0 Å). e) Crystal density of isomorphic PM7ClQ‐based crystals as a function of van der Waals volume, *V*_vdW_ of R substituents on anions.

In both fluorinated PM7ClQ‐4TFS and PM7ClQ‐TFO crystals, a large portion of fluorine‐induced interactions can be observed. As shown in **Figure** **3**a,b, the F⋅⋅⋅all atom contacts in the Hirshfeld surface fingerprint^[^ ^38^ ^]^ of anions present a relatively large portion of 27.3% and 25.3% for 4TFS and TFO anions, respectively. These interactions can result in different physical properties (e.g., molecular phonon vibrations in the THz frequency range) compared to non‐fluorinated PM7ClQ‐T crystals.

The fluorine‐induced interactions in PM7ClQ‐4TFS and PM7ClQ‐TFO crystals show both similarities and differences. As shown in Figure 3a,b, the overall pattern of Hirshfeld surface fingerprints with F⋅⋅⋅all, F⋅⋅⋅H, F⋅⋅⋅Cl, and F⋅⋅⋅C atom contacts is very similar in both crystals, which is due to the very similar molecular alignment features (Figure 2c and 2d) and the similar overall crystallographic framework (Table 1). However, the differences in the peak position, distance, and shape of the Hirshfeld surface fingerprints in Figure 3a,b for these crystals are clearly evident. Figure 3c,d shows representative F⋅⋅⋅H interactions with relatively strong interactions (distance of <3.0 Å) presented with red dotted lines in both crystals. The number and the direction of red dotted lines (F⋅⋅⋅H interactions) are remarkably different for both crystals. In addition, the number of such hydrogen bonds (F⋅⋅⋅H) with one anion is different: 3 with PM7ClQ cations and 1 with another 4TFS anion in PM7ClQ‐4TFS (Figure 3c) and 3 with PM7ClQ cations without bonds with other TFO anions in PM7ClQ‐TFO (Figure 3d).

In addition to the different interionic interactions, the space‐filling characteristics are also different in fluorinated PM7ClQ‐4TFS and PM7ClQ‐TFO and non‐fluorinated PM7ClQ‐T crystals. Although the void volume^[^ ^39^ ^]^ and the melting temperature are very similar in these three crystals, their crystal density is largely different, as listed in Table 1. The crystal density of fluorinated PM7ClQ‐4TFS and PM7ClQ‐TFO (1.431 and 1.446 gcm^−3^, respectively) is significantly higher than that of the non‐fluorinated PM7ClQ‐T (1.350 gcm^−3^). Note that the crystal density of PM7ClQ‐4TFS and PM7ClQ‐TFO is largely different than that of other PM7ClQ‐based crystals as shown in Figure 3e (e.g., PM7ClQ‐MBS). Therefore, all three crystals (PM7ClQ‐4TFS, PM7ClQ‐TFO, and PM7ClQ‐T) with fluorinated or non‐fluorinated anions exhibit different (fluorine‐induced) interionic interactions and space‐filling characteristics. These interactions affect the molecular phonon vibrations related to the THz absorption properties as well as the THz‐wave generation with these crystals, as discussed in the following section.

### 2.4 Excellent Crystal Growth Ability for High‐Performance THz Generation

To investigate the characteristics of PM7ClQ‐4TFS and PM7ClQ‐TFO in the THz frequency range, bulk single crystals were grown by solution growth methods. For bulk single‐crystal solution growth, the solubility is a very important parameter. It governs the fundamental transport of the material in the corresponding solution, which is necessary for the growth of crystals in that solution. For organic single crystals, in many cases a higher solubility results in a better bulk crystal growing ability, e.g., nonlinear optical organic crystals in the solubility range of a few grams per 100 gram solvent at ≈40 °C.^[^ ^24^, ^40^, ^41^, ^42^, ^43^ ^]^ The solubility for PM7ClQ‐4TFS and PM7ClQ‐TFO is relatively high, e.g., 1.23 and 2.31 g/100 g methanol and 2.11 and 3.82 g/100 g methanol:acetonitrile (1:1 mol/mol) (Table 1). These solubilities are higher than those of PM7ClQ‐T crystals having a lower crystal‐density (0.95 g/100 g methanol and 1.59 g/100 g methanol:acetonitrile) and also considerably higher than those of PM7ClQ‐BBS crystals having a higher crystal‐density (0.17 g/100 g methanol and 0.33 g/100 g methanol:acetonitrile).^[^ ^21^, ^22^ ^]^

**Figure 4:**
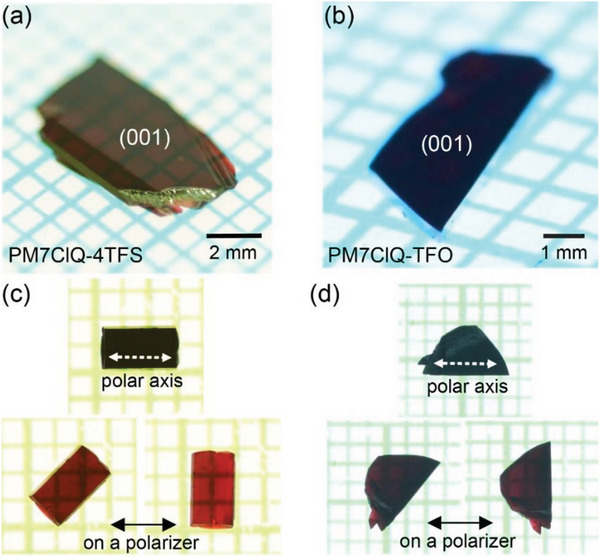
Photographs of as‐grown a) PM7ClQ‐4TFS and b) PM7ClQ‐TFO crystals. Photographs of c) a PM7ClQ‐4TFS and d) PM7ClQ‐TFO crystals on a polarizer with different crystal orientations, showing a strong optical anisotropy (higher absorption along the polar axis).

PM7ClQ‐4TFS and PM7ClQ‐TFO crystals exhibit excellent bulk crystal growth ability. **Figure** **4** shows photographs of as‐grown PM7ClQ‐4TFS and PM7ClQ‐TFO single crystals grown from methanol solvent. Both PM7ClQ‐4TFS and PM7ClQ‐TFO crystals exhibit a large area of over a few mm^2^ that presents an aperture large enough for optical experiments. In addition, both crystals possess parallel surfaces with the largest (001) facet, as determined by reflection X‐ray diffraction measurements.

To investigate THz wave characteristics of PM7ClQ‐4TFS and PM7ClQ‐TFO crystals, THz wave generation and absorption experiments were performed. We compared the properties of 0.26 mm thick PM7ClQ‐4TFS and 0.24 mm thick PM7ClQ‐TFO as‐grown crystals with (001) main facets. For comparison to the benchmark crystal, a 0.29 mm thick PM7ClQ‐T crystal with similar thickness and equivalent (001) morphology was also examined. To investigate PM7ClQ crystals as THz wave generators, a custom‐made THz time‐domain spectroscopy setup is used as described in literature.^[^ ^44^, ^45^ ^]^ The pump infrared pulses with 1.4 nJ pulse energy at 1560 nm and 38 fs pulse length at 100 MHz repetition rate were incident normally to the as‐grown (001) crystal facets with the polarization parallel to the projection of the polar axis to the (001) crystal plane. Note that in a previous report, benchmark PM7ClQ‐T crystals exhibited excellent performance in generating THz waves by optical rectification using different repetition rates in both kHz and MHz range and different THz detection methods.^[^ ^21^, ^22^ ^]^

**Figure 5:**
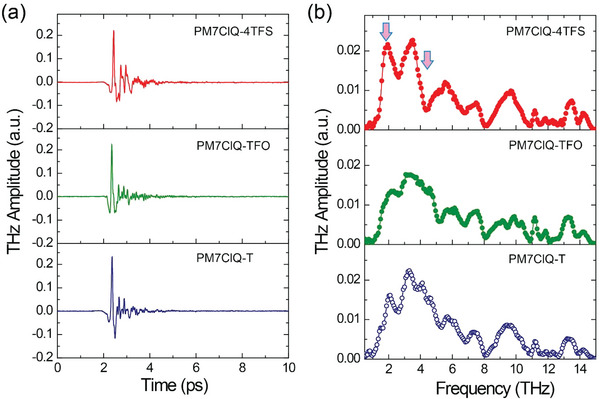
THz generation spectra of 0.26 mm thick PM7ClQ‐4TFS, 0.24 mm thick PM7ClQ‐TFO, and 0.29 mm thick PM7ClQ‐T crystals: a) time‐ and b) frequency‐domains. The spectrum in (b) presents the Fourier transform of the measured signals in (a) without additional data processing.

**Figure** **5** shows the results of the THz wave generation in 0.26 mm thick PM7ClQ‐4TFS, 0.24 mm thick PM7ClQ‐TFO, and 0.29 mm thick PM7ClQ‐T crystals in both time and frequency domain. The presented results show the directly measured signals and their Fourier transform without any corrections for geometry, filters, and detection‐crystal response. The peak‐to‐peak THz electric field of fluorinated PM7ClQ‐4TFS and PM7ClQ‐TFO crystals is comparable to benchmark non‐fluorinated PM7ClQ‐T crystal (Figure 5a). Note that PM7ClQ‐T crystals showed about one order of magnitude higher peak‐to‐peak THz electric field compared to inorganic THz crystal ZnTe in the previous report.^[^ ^21^ ^]^ This comparable peak‐to‐peak THz electric field of three PM7ClQ‐based crystals confirms the high second‐order optical nonlinearity of the fluorinated crystals, as predicted theoretically and from the SHG experiment (Figure 2e). The similar THz generation efficiency also shows that the overall phase‐matching conditions for converting 1560 nm pulses to broadband THz pulses are very similar for all crystals.

In contrast, the generated THz spectral shape produced is largely different in the three crystals (Figure 5b). For example, the generated THz amplitude of PM7ClQ‐4TFS crystal compared to PM7ClQ‐T crystal is obviously higher and lower at ≈1.9 and 4.4 THz, respectively (red arrow in Figure 5b). It is related to THz absorption characteristics as discussed below.

**Figure 6:**
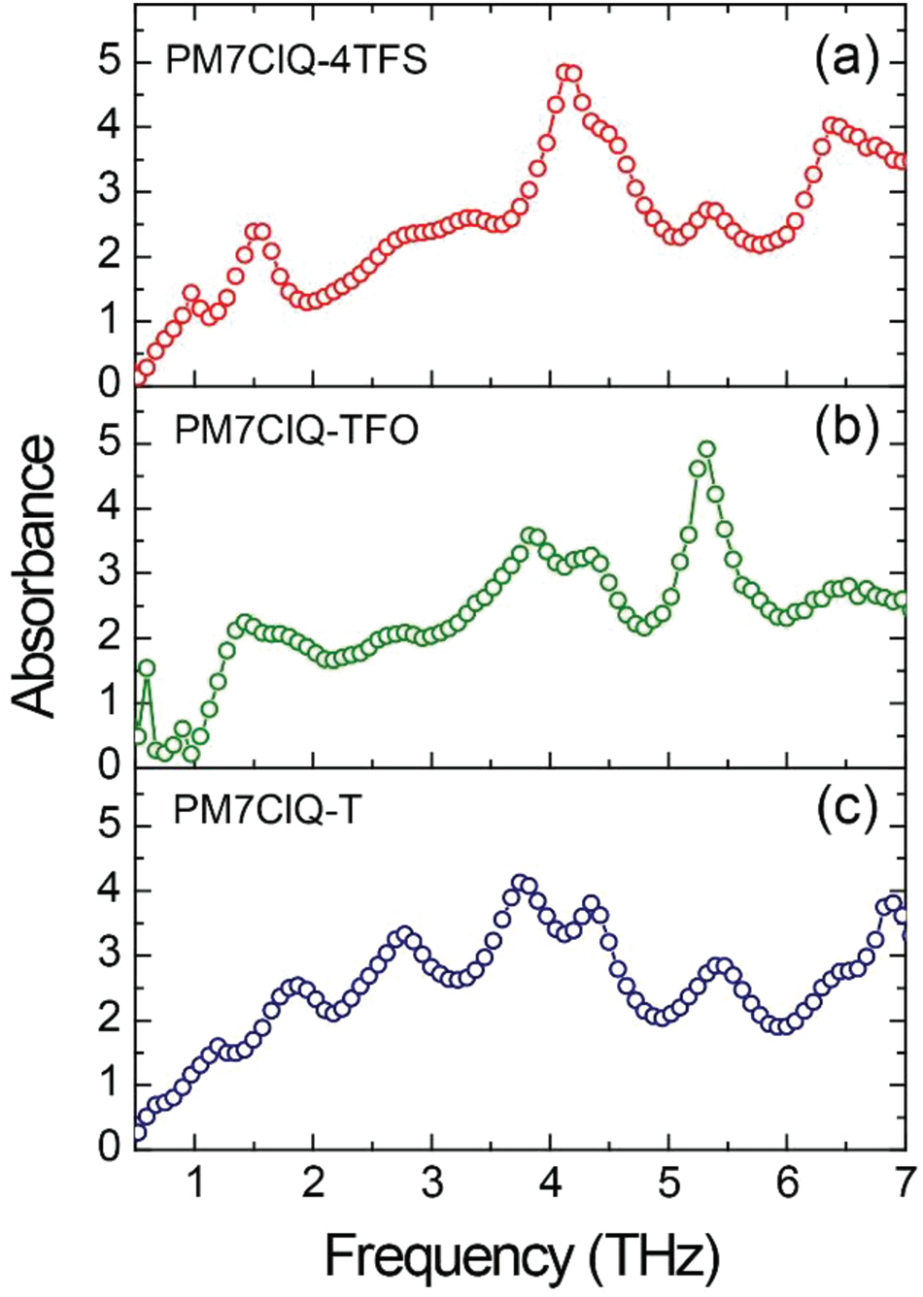
THz absorption spectra of a) 0.26 mm thick PM7ClQ‐4TFS, b) 0.24 mm thick PM7ClQ‐TFO, and c) 0.29 mm thick PM7ClQ‐T crystals.

**Figure** **6** shows the THz absorption spectra of PM7ClQ‐4TFS, PM7ClQ‐TFO, and PM7ClQ‐T crystals. The THz wave used was normally incident to the (001) surface and polarized along the projection of the polar axis to the crystal surface plane (along the white dotted arrow in Figure 4c,d), thus probing the same polarization direction as used for THz wave generation. We used a broadband THz time‐domain spectroscopy setup for THz wave generation and detection, as described in literature.^[^ ^44^, ^45^ ^]^ The spectra of these isomorphic crystals show considerably different absorption peaks and their strength. For example, the vibrational resonance at ≈5.5 THz in PM7ClQ‐T appears also in PM7ClQ‐4TFS but is slightly shifted toward lower frequencies and has an increased strength in PM7ClQ‐TFO. For PM7ClQ‐4TFS, a strong absorption resonance is observed at 4.2 THz, which is not visible in PM7ClQ‐TFO and PM7ClQ‐T or is at least considerably reduced and shifted in frequency. The well‐visible absorption resonances at ≈1.8 and 2.8 THz in PM7ClQ‐T are suppressed in both fluorinated crystals. This difference in THz absorption characteristics is attributed to different interionic interactions and different crystal densities as discussed above.

The dimple in the generated THz spectrum of PM7ClQ‐4TFS crystal at 4.4 THz (Figure 5b) originates from the absorption peak at 4.2 THz (Figure 6a). On the other hand, the dimple in the generated THz spectrum at ≈5.3 THz in PM7ClQ‐T and PM7ClQ‐TFO is absent for the generated signal in PM7ClQ‐4TFS due to the above‐mentioned suppressed absorption. Note that several dimples in the obtained THz generation spectra, overlapping for all three crystals, are due to the stronger absorption peaks in the detection DSTMS crystal (4‐*N*,*N*‐dimethylamino‐4′‐*N*’‐methylstilbazolium 2,4,6‐trimethylbenzenesulfonate, from Rainbow Photonics AG), e.g., at ≈1.0, 2.7, 6.0, and 8.3 THz,^[^ ^44^ ^]^ and not due to the investigated PM7ClQ generation crystals. These results show that despite the isomorphic crystal structure, similar nonlinear optical properties, and phase‐matching conditions, the differences in the intermolecular interactions largely affect the optical properties in the THz range that are essential for THz‐wave generation applications.

In summary, newly designed PM7ClQ‐4TFS and PM7ClQ‐TFO crystals exhibit top‐level macroscopic optical nonlinearity with the effective hyperpolarizability tensor $\beta_{iii}^{\mathrm{eff}}$ of ≥245 × 10^−30^ esu, high thermal stability with a high melting temperature of ≥255 °C (Table 1), good bulk crystal growth ability with parallel crystal facets, and a large area of over a few mm^2^ based on high solubility, and high optical‐to‐THz conversion efficiency comparable to benchmark organic crystals. In addition, although both PM7ClQ‐4TFS and PM7ClQ‐TFO crystals exhibit isomorphic crystal structure with benchmark organic crystals, additional fluorine‐induced interactions and high crystal density result in significantly different molecular phonon vibrations, which lead to different THz absorption and generation characteristics.

### 2.5 Variation of van der Waals Volume in Different Type Organic THz Crystals

**Figure 7:**
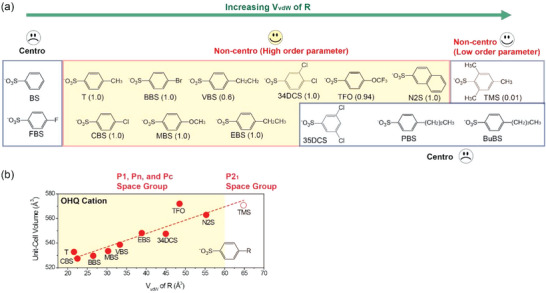
a) Crystal structure characteristics of another type of organic THz crystals, based on a cationic chromophore showing a strong tendency to form a parallel‐type cation‐anion assembly with various counter anions: the OHQ (2‐(4‐hydroxystyryl)−1‐methylquinolin‐1‐ium) cation^[^ ^46^, ^47^, ^48^, ^49^, ^50^, ^51^, ^52^ ^]^ (Non‐Centro: non‐centrosymmetric and Centro: centrosymmetric crystal structure). The number in parentheses is the corresponding order parameter. b) The unit‐cell volume (here the volume of a cation‐anion pair) of non‐centrosymmetric OHQ crystals (*Z* = 1 and ≠ 1) from Figure 7a, as a function of van der Waals volume, *V*_vdW_ of R substituents^[^ ^29^, ^30^ ^]^ on anions with the line slope of 1.09.

To confirm more clearly our proposal to control the van der Waals volume for achieving non‐centrosymmetric crystal structure of highly polar cationic chromophores, **Figure** **7** shows the crystal structure characteristics of previously reported benchmark organic THz crystals that exhibit a different type of cation‐anion assembly compared to PM7ClQ‐based crystals in this work. Many organic THz salt crystals can be classified into two different types of cation‐anion assembly: the series type and the parallel type.^[^ ^20^ ^]^ The nonlinear optical OHQ (2‐(4‐hydroxystyryl)−1‐methylquinolin‐1‐ium) cationic chromophore forms a non‐centrosymmetric crystal structure with various benzenesulfonate anions.^[^ ^46^, ^47^, ^48^, ^49^, ^50^, ^51^, ^52^ ^]^ While PM7ClQ‐based crystals exhibit a series‐type cation‐anion assembly, OHQ‐based crystals show a strong tendency to form a parallel cation‐anion assembly.

With varying the der Waals volumes of R substituents on anions in OHQ‐based crystals, the trend of the resulting crystal symmetry (Figure 7a) as well as the approximately linear relationship with the unit‐cell volume (here the volume of a cation‐anion pair because of OHQ‐based crystals with *Z ≠* 1, Figure 7b) are similar with those of PM7ClQ‐based crystals (Figure 1b–d). However, the resulting crystal space group symmetry of OHQ‐based crystals largely varies, including the triclinic *P1* and monoclinic *Pn, Pc*, and *P2*_1_ space groups (Figure 7b). In addition, the order parameters also largely vary; see the numbers in parentheses in Figure 7a. Note that compared to OHQ‐based crystals, other parallel‐type crystals even more sensitively change the crystal structure characteristics with varying the van der Waals volume of substituents and often exhibit large deviations from the results achieved in PM7ClQ‐based crystals.^[^ ^11^, ^20^ ^]^ However, it is clear that introducing a certain range of van der Waals volumes of anions for many specific cationic chromophores shows a strong tendency to form non‐centrosymmetric crystal structure in the crystalline state and leads to large macroscopic optical nonlinearities.

## 3 Conclusion

We have successfully demonstrated a rational design of high‐performance organic nonlinear optical and THz generation crystals (PM7ClQ‐4TFS and PM7ClQ‐TFO) by optimization of van der Waals volume and shape of molecular anions, but keeping the favorable overall crystallographic framework (i.e., main supramolecular interactions including Coulomb interactions and overall crystallographic parameters) of benchmark crystals. Introducing molecular ions within a certain range of van der Waals volume results in the predicted crystal structure with the desired non‐centrosymmetric molecular ordering of PM7ClQ chromophores in crystals, showing large effective hyperpolarizability tensor element $\beta_{iii}^{\mathrm{eff}}$ (≥245 × 10^−30^ esu). In addition, when increasing the van der Waals volume of anions, the crystal structure consistently changes. Newly designed fluorinated PM7ClQ‐4TFS and PM7ClQ‐TFO crystals exhibit excellent THz wave generation but show distinctively different THz spectrum and absorption characteristics compared to benchmark crystals. Therefore, controlling the van der Waals volume and the shape of the molecular ionic components is a highly potential design strategy toward solving the unfailingly enigmatic question of achieving non‐centrosymmetric crystal structure in organic crystals that are required in diverse applications such as nonlinear optics, electro‐optics, THz photonics, and ferroelectrics.

## 4 Experimental Section

The details of synthesis and crystal characteristics of newly designed crystals are described in Supporting Information.

## Conflict of Interest

The authors declare no conflict of interest.

## Supporting information

Supporting Information

## Data Availability

The data that support the findings of this study are available from the corresponding author upon reasonable request.
