# Supplementary material for: Design of High‐Performance Organic Nonlinear Optical and Terahertz Crystals by Controlling the van der Waals Volume
Source: Adv Sci (Weinh). 2023 Oct 22;10(34):2304767. doi: 10.1002/advs.202304767 (PMC10700226; doi:10.1002/advs.202304767)
Supplement: Supplementary file 1 — Supporting Information [file ADVS-10-2304767-s001.pdf]

ADVANCED SCIENCE
Open Access

Supporting Information

for *Adv. Sci.*, DOI 10.1002/advs.202304767

Design of High-Performance Organic Nonlinear Optical and Terahertz Crystals by Controlling the van der Waals Volume

Bong-Rim Shin, Uros Puc, Yu-Jin Park, Dong-Joo Kim, Chae-Won Lee, Woojin Yoon, Hoseop Yun, Chaeyoon Kim, Fabian Rotermund, Mojca Jazbinsek and O-Pil Kwon**

Supporting Information

Design of High-Performance Organic Nonlinear Optical and Terahertz Crystals by Controlling the van der Waals Volume

Bong-Rim Shin[†]*, Uros Puc*[†]*, Yu-Jin Park*[†]*, Dong-Joo Kim, Chae-Won Lee, Woojin Yoon, Hoseop Yun, Chaeyoon Kim, Fabian Rotermund, Mojca Jazbinsek*, O-Pil Kwon**

((Optional Dedication))

B. R. Shin, Y. J. Park, D. J. Kim, C. W. Lee, Prof. O. P. Kwon
Department of Molecular Science and Technology, Ajou University, Suwon 16499 (Korea)
E-mail: opilkwon@ajou.ac.kr
Dr. U. Puc, Dr. M. Jazbinsek
Institute of Computational Physics, Zurich University of Applied Sciences (ZHAW), 8401 Winterthur (Switzerland)
E-mail: mojca.jazbinsek@zhaw.ch
C. Kim, Prof. F. Rotermund
Department of Physics, Korea Advanced Institute of Science and Technology (KAIST), Daejeon 34141 (Korea)
Dr. W. Yoon, Prof. H. Yun
Research Institute of Basic Sciences, Department of Chemistry, Department of Energy Systems Research, Ajou University, Suwon 16499 (Korea)

[†]These authors contributed equally to this work.

A. Synthesis

The synthesis of PM7ClQ-based salt compounds with different anions (PM7ClQ-BS, PM7ClQ-FBS, PM7ClQ-4TFS, PM7ClQ-3TFS, PM7ClQ-TFO, PM7ClQ-N2S, and PM7ClQ-N1S) is based on the condensation reaction between 4-(4-(hydroxymethyl)piperidin-1-yl)benzaldehyde and the corresponding intermediates, similar to the literature. [*Adv. Optic. Mater.*, **2021**, *9*, 2100324]

7-Chloro-2-(4-(4-(hydroxymethyl)piperidin-1-yl)styryl)-1-methylquinolin-1-ium benzenesulfonate (PM7ClQ-BS): Yield = 3.7 %. ^{1}H NMR (600 MHz, DMSO-d_6, δ): 8.81 (d, 1H, *J* = 9.64 Hz, $C_{10}H_8ClN$), 8.59 (s, 1H, $C_{10}H_8ClN$), 8.52 (d, 1H, *J* = 8.95 Hz, $C_{10}H_8ClN$), 8.27 (m, 2H, C_2H_2 ,$C_{10}H_8ClN$), 7.92 (m, 1H, $C_{10}H_8ClN$), 7.86 (d, 2H, *J* = 8.95 Hz, C_6H_4), 7.58 (m, 3H, C_2H_2, $C_6H_4SO_3^-$), 7.31 (m, 3H, $C_6H_4SO_3^-$), 7.07 (s, 2H, *J* = 8.95 Hz, C_6H_4), 4.54 (t, 1H, *J* = 5.16 Hz, OH), 4.41 (s, 3H, NCH_3), 4.07 (d, 2H, *J* = 11.71 Hz, CH_2OH), 3.28 (t, 2H, *J* = 5.51 Hz, C_5H_9N), 2.93 (t, 2H, *J* = 11.71 Hz, C_5H_9N), 1.76 (d, 2H, *J* = 14.46 Hz, C_5H_9N), 1.67 (m, 1H, C_5H_9N), 1.17 (m, 2H, C_5H_9N). ^{13}C NMR (DMSO-d_6, δ): 156.91, 153.18, 149.34, 148.33, 141.53, 139.90, 139.03, 132.35, 131.52, 128.52, 127.73, 125.60, 125.56, 123.37, 120.59, 118.64, 113.66, 112.26, 65.64, 46.76, 38.29, 28.15. Elemental analysis of $C_{30}H_{31}ClN_2O_4S$: calc'd. C 65.38, H 5.67, Cl 6.43, N 5.08, O 11.61, S 5.82; found: C 65.58, H 5.73, N 5.30, S 5.77.

7-Chloro-2-(4-(4-(hydroxymethyl)piperidin-1-yl)styryl)-1-methylquinolin-1-ium 4-fluorobenzenesulfonate (PM7ClQ-FBS):. Yield = 54.7 %. ^{1}H NMR (600 MHz, DMSO-d_6, δ): 8.80 (d, 1H, *J* = 9.6 Hz, $C_{11}H_{11}ClN$), 8.58 (s, 1H, $C_{11}H_{11}ClN$), 8.52 (d, 1H, *J* = 9 Hz, $C_{11}H_{11}ClN$), 8.26 (m, 2H, C_2H_2 ,$C_{11}H_{11}ClN$), 7.92 (d, 1H, *J* = 8.3 Hz, $C_{11}H_{11}ClN$), 7.86 (d, 2H, *J* = 9 Hz, C_6H_4), 7.61 (m, 2H, $C_6H_4SO_3^-$), 7.57 (d,1H, *J* = 15.2 Hz, C_2H_2), 7.12 (m, 2H, $C_6H_4SO_3^-$), 7.06 (d, 2H, *J* = 8.3 Hz, C_6H_4), 4.52 (t, 1H, *J* = 5.2 Hz, OH), 4.41 (s, 3H, NCH_3), 4.07 (d, 2H, *J* = 13.1 Hz, CH_2OH), 3.29 (m, 2H, C_5H_9N), 2.93 (m, 2H, C_5H_9N), 1.76 (m, 2H, C_5H_9N), 1.67 (m, 1H, C_5H_9N), 1.19 (m, 2H, C_5H_9N). ^{13}C NMR (DMSO-d_6, δ): 163.21,

161.59, 157.48, 153.75, 149.89, 145.55, 142.04, 140.46, 139.51, 132.86, 131.99, 129.04, 128.35, 126.11, 123.91, 121.09, 119.20, 114.85, 114.23, 112.83, 66.09, 47.29, 38.79, 28.62. Elemental analysis of $C_{30}H_{30}ClFN_2O_4S$: calc'd. C 63.32, H 5.31, Cl 6.23, F 3.34, N 4.92, O 11.25, S 5.63; found: C 63.65, H 5.28, N 4.94, S 5.64.

7-Chloro-2-(4-(4-(hydroxymethyl)piperidin-1-yl)styryl)-1-methylquinolin-1-ium 4-(trifluoromethyl)benzenesulfonate (PM7ClQ-4TFS): Yield = 50 %. ^{1}H NMR (600 MHz, DMSO-d_6, δ): 8.80 (d, 1H, J = 9 Hz, $C_{11}H_{11}ClN$), 8.58 (s, 1H, $C_{11}H_{11}ClN$), 8.52 (d, 1H, J = 9.6 Hz, $C_{11}H_{11}ClN$), 8.27 (m, 1H, C_2H_2), 8.27 (m, 1H, $C_{11}H_{11}ClN$), 7.91 (d, 1H, J = 8.4 Hz, $C_{11}H_{11}ClN$), 7.86 (d, 2H, J = 8.4 Hz, C_6H_4), 7.79 (d, 2H, J = 8.4 Hz, $C_6H_4SO_3^-$), 7.70 (d, 2H, J = 8.4 Hz, $C_6H_4SO_3^-$), 7.57 (d, 1H, J = 15 Hz, C_2H_2), 7.06 (d, 2H, J = 9.6 Hz, C_6H_4), 4.52 (t, 1H, J = 4.8 Hz, OH), 4.41 (s, 3H, NCH_3), 4.07 (m, 2H, CH_2OH), 3.29 (m, 2H, C_5H_9N), 2.93 (m, 2H, C_5H_9N), 1.76 (m, 2H, C_5H_9N), 1.67 (m, 1H, C_5H_9N), 1.19 (m, 2H, C_5H_9N). ^{13}C NMR (DMSO-d_6, δ): 156.94, 153.21, 152.22, 149.34, 141.50, 139.92, 138.97, 132.32, 131.46, 128.74, 128.51, 126.87, 126.33, 125.58, 125.07, 124.89, 123.37, 123.26, 121.46, 120.55, 118.66, 113.68, 112.29, 65.56, 46.75, 38.27, 28.10. Elemental analysis of $C_{31}H_{30}ClF_3N_2O_4S$: calc'd. C 60.14, H 4.88, Cl 5.73, F 9.21, N 4.52, O 10.34, S 5.18; found: C 60.39, H 4.92, N 4.58, S 4.99.

7-Chloro-2-(4-(4-(hydroxymethyl)piperidin-1-yl)styryl)-1-methylquinolin-1-ium 3-(trifluoromethyl)benzenesulfonate (PM7ClQ-3TFS): Yield = 63 %. ^{1}H NMR (600 MHz, DMSO-d_6, δ): 8.80 (d, 1H, J = 9 Hz, $C_{11}H_{11}ClN$), 8.58 (s, 1H, $C_{11}H_{11}ClN$), 8.52 (d, 1H, J = 9.6 Hz, $C_{11}H_{11}ClN$), 8.27 (m, 1H, C_2H_2), 8.27 (m, 1H, $C_{11}H_{11}ClN$), 7.91 (d, 1H, J = 8.1 Hz, $C_{11}H_{11}ClN$), 7.86 (m, 1H, $C_6H_4SO_3^-$), 7.86 (m, 1H, $C_6H_4SO_3^-$), 7.86 (m, 2H, C_6H_4), 7.69 (d, 1H, J = 7.8 Hz, $C_6H_4SO_3^-$), 7.58 (m, 1H, $C_6H_4SO_3^-$), 7.58 (m, 1H, C_2H_2), 7.06 (d, 2H, J = 10.2 Hz, C_6H_4), 4.50 (t, 1H, J = 5.4 Hz, OH), 4.42 (s, 3H, NCH_3), 4.07 (m, 2H, CH_2OH), 3.29 (m, 2H, C_5H_9N), 2.93 (m, 2H, C_5H_9N), 1.76 (m, 2H, C_5H_9N), 1.67 (m, 1H, C_5H_9N), 1.19 (m, 2H, C_5H_9N). ^{13}C NMR (DMSO-d_6, δ): 156.94, 153.21, 149.43, 149.33, 141.50, 139.91, 138.97,

132.30, 131.45, 129.52, 129.16, 128.50, 128.45, 126.80, 125.57, 125.09, 125.00, 123.37, 123.19, 121.93, 121.39, 120.55, 118.64, 113.68, 112.28, 65.54, 46.74, 38.24, 28.08. Elemental analysis of $C_{31}H_{30}ClF_3N_2O_4S$: calc'd. C 60.14, H 4.88, Cl 5.73, F 9.21, N 4.52, O 10.34, S 5.18; found: C 60.91, H 4.95, N 4.62, S 5.24.

7-Chloro-2-(4-(4-(hydroxymethyl)piperidin-1-yl)styryl)-1-methylquinolin-1-ium 4-(trifluoromethoxy)benzenesulfonate (PM7ClQ-TFO): Yield = 34.7 %. ^{1}H NMR (600 MHz, DMSO-d_6, δ): 8.80 (d, 1H, J = 8.95 Hz, $C_{10}H_8ClN$), 8.58 (s, 1H, $C_{10}H_8ClN$), 8.52 (d, 1H, J = 8.95 Hz, $C_{10}H_8ClN$), 8.27 (m, 2H, C_2H_2 ,$C_{10}H_8ClN$), 7.91 (d, 1H, J = 8.26 Hz, $C_{10}H_8ClN$), 7.86 (d, 2H, J = 8.95 Hz, C_6H_4), 7.69 (d, 2H, J = 8.95 Hz, $C_6H_4SO_3^-$), 7.57 (d, 1H, J = 15.15 Hz, C_2H_2), 7.30 (d, 2H, J = 8.26 Hz, $C_6H_4SO_3^-$), 7.06 (d, 2H, J = 8.95 Hz, C_6H_4), 4.51 (t, 1H, J = 5.16 Hz, OH), 4.41 (s, 3H, NCH_3), 4.07 (d, 2H, J = 13.08 Hz, CH_2OH), 3.29 (m, 2H, C_5H_9N), 2.93 (m, 2H, C_5H_9N), 1.76 (m, 2H, C_5H_9N), 1.67 (s, 1H, C_5H_9N), 1.18 (m, 2H, C_5H_9N). ^{13}C NMR (DMSO-d_6, δ): 156.89, 153.17, 149.33, 148.12, 147.66, 141.47, 139.88, 138.96, 132.29, 131.46, 128.47, 127.65, 125.56, 123.35, 122.58, 120.89, 120.53, 120.25, 119.12, 118.62, 117.49, 113.63, 112.24, 65.56, 46.72, 38.25, 28.09. Elemental analysis of $C_{31}H_{30}ClF_3N_2O_4S$: calc'd. C 58.63, H 4.76, Cl 5.58, F 8.97, N 4.41, O 12.60, S 5.05; found: C 58.61, H 4.96, N 4.72, S 5.07.

7-Chloro-2-(4-(4-(hydroxymethyl)piperidin-1-yl)styryl)-1-methylquinolin-1-ium naphthalene-2-sulfonate (PM7ClQ-N2S): Yield = 40 %. ^{1}H NMR (600 MHz, DMSO-d_6, δ): 8.80 (d, 1H, J = 9 Hz, $C_{11}H_{11}ClN$), 8.58 (s, 1H, $C_{11}H_{11}ClN$), 8.52 (d, 1H, J = 9.6 Hz, $C_{11}H_{11}ClN$), 8.27 (m, 1H, C_2H_2), 8.27 (m, 1H, $C_{11}H_{11}ClN$), 8.13 (s, 1H, $C_{10}H_7SO_3^-$), 7.97 (m, 1H, $C_{10}H_7SO_3^-$), 7.90 (m, 1H, $C_{11}H_{11}ClN$), 7.90 (m, 1H, $C_{10}H_7SO_3^-$), 7.85 (m, 1H, $C_{10}H_7SO_3^-$), 7.85 (m, 2H, C_6H_4), 7.70 (d, 1H, J = 8.4 Hz, $C_{10}H_7SO_3^-$), 7.57 (d, 1H, J = 15 Hz, C_2H_2), 7.52 (m, 1H, $C_{10}H_7SO_3^-$), 7.52 (m, 1H, $C_{10}H_7SO_3^-$), 7.06 (d, 2H, J = 9 Hz, C_6H_4), 4.50 (t, 1H, J = 5.4 Hz, OH), 4.42 (s, 3H, NCH_3), 4.07 (m, 2H, CH_2OH), 3.29 (m, 2H, C_5H_9N), 2.93 (m, 2H, C_5H_9N), 1.76 (m, 2H, C_5H_9N), 1.67 (m, 1H, C_5H_9N), 1.19 (m, 2H, C_5H_9N). ^{13}C NMR

(DMSO-d_6, δ): 156.94, 153.20, 149.32, 145.72, 141.49, 139.89, 138.96, 132.67, 132.31, 132.14, 131.45, 128.49, 128.41, 127.42, 127.24, 126.35, 126.25, 125.56, 124.01, 123.99, 123.37, 120.55, 118.63, 113.68, 112.29, 65.54, 46.74, 38.24, 28.08. Elemental analysis of $C_{34}H_{33}ClN_2O_4S$: calc'd. C 67.93, H 5.53, Cl 5.90, N 4.66, O 10.65, S 5.33; found: C 68.39, H 5.51, N 4.79, S 5.35.

7-Chloro-2-(4-(4-(hydroxymethyl)piperidin-1-yl)styryl)-1-methylquinolin-1-ium naphthalene-1-sulfonate (PM7ClQ-N1S): Yield = 41 %. ^{1}H NMR (600 MHz, DMSO-d_6, δ): 8.84 (m, 1H, $C_{10}H_7SO_3^-$), 8.80 (d, 1H, J = 9.6 Hz, $C_{11}H_{11}ClN$), 8.58 (s, 1H, $C_{11}H_{11}ClN$), 8.51 (d, 1H, J = 9.6 Hz, $C_{11}H_{11}ClN$), 8.27 (m, 1H, C_2H_2), 8.27 (m, 1H, $C_{11}H_{11}ClN$), 7.89 (m, 1H, $C_{10}H_7SO_3^-$), 7.89 (m, 1H, $C_{11}H_{11}ClN$), 7.89 (m, 1H, $C_{10}H_7SO_3^-$), 7.89 (m, 1H, $C_{10}H_7SO_3^-$), 7.89 (m, 2H, C_6H_4), 7.57 (d, 1H, J = 15 Hz, C_2H_2), 7.49 (m, 1H, $C_{10}H_7SO_3^-$), 7.49 (m, 1H, $C_{10}H_7SO_3^-$), 7.42 (t, 1H, J = 7.2 Hz, $C_{10}H_7SO_3^-$), 7.06 (d, 2H, J = 9 Hz, C_6H_4), 4.51 (t, 1H, J = 6 Hz, OH), 4.41 (s, 3H, NCH_3), 4.07 (m, 2H, CH_2OH), 3.29 (m, 2H, C_5H_9N), 2.93 (m, 2H, C_5H_9N), 1.76 (m, 2H, C_5H_9N), 1.67 (m, 1H, C_5H_9N), 1.19 (m, 2H, C_5H_9N). ^{13}C NMR (DMSO-d_6, δ): 156.87, 153.15, 149.29, 144.10, 141.45, 139.84, 138.94, 133.50, 132.27, 131.43, 129.28, 129.04, 128.44, 127.67, 125.52, 125.47, 124.41, 124.29, 123.33, 120.51, 118.57, 113.61, 112.22, 65.55, 46.71, 38.22, 28.07. Elemental analysis of $C_{34}H_{33}ClN_2O_4S$: calc'd. C 67.93, H 5.53, Cl 5.90, N 4.66, O 10.65, S 5.33; found: C 68.45, H 5.53, N 4.79, S 5.31.

B. Crystal Structure of PM7ClQ-4TFS and PM7ClQ-TFO

PM7ClQ-4TFS: The PM7ClQ-4TFS single crystals were grown by a rapid cooling method in methanol:acetonitrile (1:1 mol/mol) mixture. $C_{24}H_{26}ClN_2O{\cdot}C_7H_4F_3O_3S$, M_r = 619.08, triclinic, space group *P*1, *a* = 7.0905(5) Å, *b* = 10.3016(7) Å, *c* = 11.0434(9) Å, α = 66.469(2)°, β = 76.744(1)°, γ = 81.248(2)°, *V* = 718.20(9) Å^3, *Z* = 1, *T* = 290(1) K, μ(MoKα)= 0.27 mm^{-1}. Of 7039 reflections collected in the θ range 3.3°-27.5° using ω scans on a Rigaku R-axis Rapid S diffractometer, 5325 were unique reflections (R_{int} = 0.023). The structure was solved and refined against F^2 using SHELXL-2018/3, [G. M. Sheldrick, *Acta Cryst*., C71, 3 (2015)] 381 variables, wR_2 = 0.127, R_1 = 0.040 ($F_o^2 > 2\sigma(F_o^2)$), GOF = 1.10, and max/min residual electron density 0.33/-0.29 eÅ^{-3}. CCDC-2070575.

PM7ClQ-TFO: The PM7ClQ-TFO single crystals were grown by a slow cooling method in methanol. $C_{24}H_{26}ClN_2O{\cdot}C_7H_4F_3O_4S$, M_r = 635.08, triclinic, space group *P*1, *a* = 7.0759 (4) Å, *b* = 10.3428 (9) Å, *c* = 10.8490 (7) Å, α = 71.427 (3)°, β = 76.025 (1)°, γ = 82.894 (3)°, *V* = 729.41 (9) Å^3, *Z* = 1, *T* = 290 (1) K, μ(MoKα)= 0.27 mm^{-1}. Of 7096 reflections collected in the θ range 3.3 ~ 27.5° using ω scans on a Rigaku R-axis Rapid S diffractometer, 5370 were unique reflections (R_{int} = 0.045). The structure was solved and refined against F^2 using SHELXL-2018/3, [G. M. Sheldrick, *Acta Cryst*., C71, 3 (2015)] 390 variables, wR_2 = 0.200, R_1 = 0.063 ($F_o^2 > 2\sigma(F_o^2)$), GOF = 1.08, and max/min residual electron density 0.59/-0.31 e Å^{-3}. CCDC-2209710.

C. Polymorphism of PM7ClQ-FBS

PM7ClQ-FBS exhibits a polymorphism with two polymorphs (Phase-I and -II) found in this work. The first phase, PM7ClQ-FBS (Phase-I) was obtained by a fast-cooling solution growth method (≤ 1 ^{o}C/h) from two different solvents and exhibits a strong SHG signal, as shown in Figure S1a and S1b. This demonstrates that PM7ClQ-FBS (Phase-I) possesses a non-centrosymmetric molecular ordering in the crystalline state. The second phase, PM7ClQ-FBS (Phase-II) was obtained by a slow-cooling solution growth method (1 ^{o}C/day) from methanol:acetonitrile (1:1 mol/mol) mixture. PM7ClQ-FBS (Phase-II) exhibits centrosymmetric molecular ordering with monoclinic $P2_1/c$ space group (CCDC-2209705). The details of the crystal structure analysis of PM7ClQ-FBS (Phase-II) are described below and the corresponding molecular alignment is shown in Figure S2. In this phase, there is a different type of cation-anion assembly observed as for the anions in the optimal range of the van der Waals volumes.

While as-grown PM7ClQ-FBS (Phase-II) single crystals (e.g., Figure S2a) have a high enough quality and size for reliable X-ray single crystal structure analysis, as-grown PM7ClQ-FBS (Phase-I) single crystals (e.g., Figure S1c) have not. To investigate crystal characteristics of PM7ClQ-FBS (Phase-I), we measured powder X-ray diffraction patterns of PM7ClQ-FBS (Phase-I) and compared them to the known PM7ClQ-T with *P*1 space group (CCDC-1950430). As shown in Figure S1d, the X-ray diffraction pattern of PM7ClQ-FBS (Phase-I) is very similar to that of PM7ClQ-T and its crystal structure. Consequently, PM7ClQ-FBS (Phase-I) possibly exhibits an isomorphic or at least a pseudo-isomorphic crystal structure with *P*1 space group of PM7ClQ-T crystals (Pseudo-*P*1 in Figure 1b). This polymorphism shows that the FBS anion is located just at the border of the optimal range of the van der Waals volumes of anions illustrated in Figure 1.

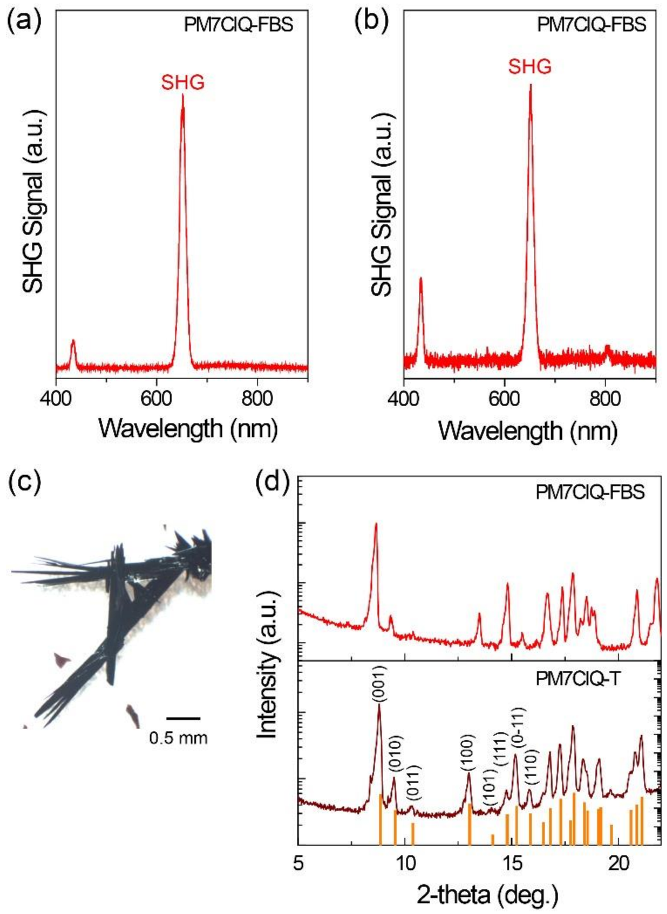

Figure S1. PM7ClQ-FBS (Phase-I). (a-b) Powder second harmonic generation (SHG) measurement at 1300 nm of PM7ClQ-FBS (Phase-I) powders crystallized with a relatively fast cooling rate (≤ 1 ^{o}C/h) in (a) methanol and (b) methanol:acetonitrile (1:1 mol/mol) mixture. (c) Photographs of as-grown PM7ClQ-FBS (Phase-I) single crystals by slow cooling method with a cooling rate of 1 ^{o}C/h in methanol:acetonitrile (1:1 mol/mol) mixture. (d) Powder X-ray diffraction patterns (logarithmic intensity scale) of PM7ClQ-FBS (Phase-I) and PM7ClQ-T powders recrystallized in identical solvent, methanol. The orange bars present the diffraction peaks of the single crystal structure of PM7ClQ-T with *P*1 space group (CCDC-1950430).

PM7ClQ-FBS (Phase-II) crystal structure: The PM7ClQ-FBS single crystals were grown by a slow cooling method with a cooling rate of 1 ^{o}C/day in methanol:acetonitrile (1:1 mol/mol) mixture. $C_{24}H_{26}ClN_2O{\cdot}C_6H_4FO_3S$, M_r = 569.07, monoclinic, space group $P2_1/c$, a = 10.9820 (4) Å, b = 19.2031 (6) Å, c = 13.2768 (5) Å, β = 96.782 (1)°, V = 2780.33 (17) Å^3, Z = 4, T = 290 (1) K, μ(MoKα)= 0.26 mm^{-1}. Of 26939 reflections collected in the θ range 3.1 ~ 27.5° using ω scans on a Rigaku R-axis Rapid S diffractometer, 6343 were unique reflections (R_{int} = 0.017). The structure was solved and refined against F^2 using SHELXL-2018/3, [G. M. Sheldrick, *Acta Cryst*., C71, 3 (2015)] 354 variables, wR_2 = 0.136, R_1 = 0.043 ($F_o^2 > 2\sigma(F_o^2)$), GOF = 1.10, and max/min residual electron density 0.49/-0.36 e Å^{-3}. CCDC-2209705.

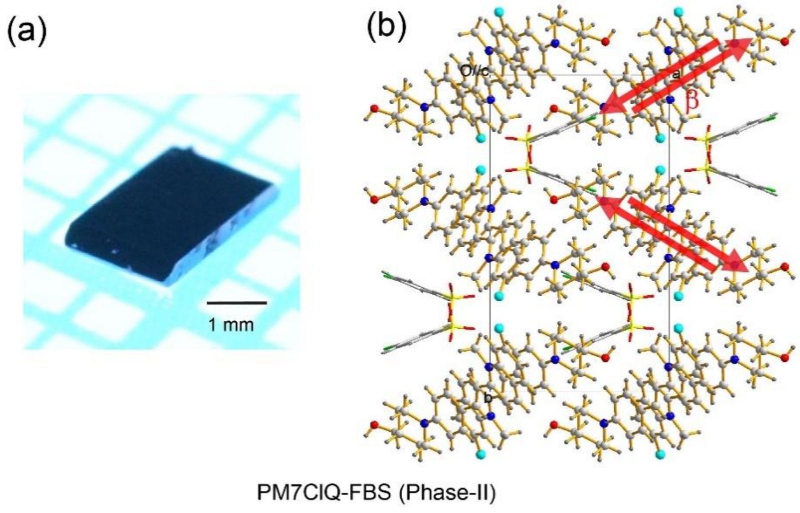

Figure S2. PM7ClQ-FBS (Phase-II). (a) Photographs of as-grown PM7ClQ-FBS (Phase-II) single crystal obtained by a slow cooling method with a cooling rate of 1 ^{o}C/day in methanol:acetonitrile (1:1 mol/mol) mixture. (b) Molecular ordering of PM7ClQ-FBS (Phase-II) exhibiting centrosymmetric $P2_1/c$ space group symmetry (CCDC-2209705).
